# Distribution of Transferable Antibiotic Resistance Genes in Laboratory-Reared Edible Mealworms (*Tenebrio molitor* L.)

**DOI:** 10.3389/fmicb.2018.02702

**Published:** 2018-11-19

**Authors:** Andrea Osimani, Vesna Milanović, Federica Cardinali, Cristiana Garofalo, Francesca Clementi, Sara Ruschioni, Paola Riolo, Nunzio Isidoro, Nino Loreto, Roberta Galarini, Simone Moretti, Annalisa Petruzzelli, Eleonora Micci, Franco Tonucci, Lucia Aquilanti

**Affiliations:** ^1^Dipartimento di Scienze Agrarie, Alimentari ed Ambientali, Università Politecnica delle Marche, Ancona, Italy; ^2^Istituto Zooprofilattico Sperimentale dell'Umbria e delle Marche, Perugia, Italy; ^3^Istituto Zooprofilattico Sperimentale dell'Umbria e delle Marche, Centro di Riferimento Regionale Autocontrollo, Pesaro, Italy

**Keywords:** novel foods, edible insects, metagenomic DNA analysis, nested-PCR, insect resistome

## Abstract

In the present study, the distribution of antibiotic resistance genes in laboratory-reared fresh mealworm larvae (*Tenebrio molitor* L.), their feeding substrates (carrots and wheatmeal), and frass was assessed. Microbial counts on selective media added with antibiotics highlighted the presence of lactic acid bacteria resistant to ampicillin and vancomycin and, more specifically, enterococci resistant to the latter antibiotic. Moreover, staphylococci resistant to gentamicin, erythromycin, tetracycline, and vancomycin were detected. Enterobacteriaceae resistant to ampicillin and gentamicin were also found, together with Pseudomonadaceae resistant to gentamicin. Some of the genes coding for resistance to macrolide-lincosamide-streptogramin B (MLS_B_) [*erm*(A), *erm*(C)], vancomycin [*vanA, vanB*], tetracycline [*tet*(O)], and β-lactams [*mecA* and *blaZ*] were absent in all of the samples. For the feeding substrates, organic wheatmeal was positive for *tet*(S) and *tet*(K), whereas no AR genes were detected in organic carrots. The genes *tet*(M), *tet*(K), and *tet*(S) were detected in both mealworms and frass, whereas gene *aac-aph*, coding for resistance to amynoglicosides was exclusively detected in frass. No residues for any of the 64 antibiotics belonging to 10 different drug classes were found in either the organic wheatmeal or carrots. Based on the overall results, the contribution of feed to the occurrence of antibiotic resistance (AR) genes and/or antibiotic-resistant microorganisms in mealworm larvae was hypothesized together with vertical transmission via insect egg smearing.

## Introduction

“*Them insects eats up every blessed green thing that do grow, and us farmers starves. Well, eat them, and grow fat”* (Vincent M. Holt). In 1885, Vincent M. Holt published a small brochure, titled “*Why not eat insects?*” in which he explained his theory of “entomophagy,” referring to the human practice of eating insects. The pamphlet, which was reprinted in 1995, ended with some interesting recipes for insect-based menus (Holt, 1995). At present, the eccentric nature of the above-mentioned text should be considered more closely. Insects are widely consumed in Asia, Africa, and South America, where they constitute part of the traditional diets of at least 2 billion people. By contrast, in Europe, they are rarely consumed and are often associated with low prestige and poor countries (van Huis et al., 2013; Verbeke, 2015). Nevertheless, insects can provide an alternative source of high-quality protein and nutritionally valuable substances (e.g., good lipids, micronutrients, B-group vitamins, and fibers) that are exploitable by the food industry (Schlüter et al., 2016).

As reported by van Huis (2016), in a few European countries, such as Belgium, the Netherlands, Austria, and France, the production and consumption of processed edible insects is tolerated in some ways, whereas in Switzerland, it is allowed. In these countries, the rearing of insects for both human and animal consumption is mainly carried out by small private enterprises, but the edible insect sector is rapidly growing; thus, the need for specific food laws to regulate insect rearing and processing is increasingly urgent (van Huis, 2016).

Recently, the European Union issued Regulation (EU) No 2015/2283 of the European Parliament and the Council of 25 November 2015 on novel foods that will go into effect 1 January 2018, introducing a simplified and centralized procedure for introducing edible insects into the EU market. As novel foods, the safety of edible insects must be assessed before they can be considered safe, since insects can be a source of toxic substances and pathogenic microorganisms. As reported by Schlüter et al. (2016), there is still a lack of knowledge regarding the risks associated with the use of insects in the production of foods and food ingredients.

Regarding the microbiological risks, several studies have already been focused on the microbiota of edible insects through conventional microbiological analyses and advanced DNA-based techniques (Simpanya et al., 2000; Banjo et al., 2006; Klunder et al., 2012; Stoops et al., 2016; Garofalo et al., 2017; Osimani et al., 2017a; Vandeweyer et al., 2017). The results of these studies highlighted the presence of potential human pathogens, suggesting the need for a deeper investigation of the correlation between the microbiota and insect rearing conditions.

In addition to opportunistic human pathogens, even bacteria harboring transferable antibiotic resistance (AR) genes pose a serious threat to human health. The emergence and spread of transferable AR is a global public health problem that requires action across all government sectors and society. As reported by the World Health Organization (WHO), new AR mechanisms are constantly emerging and spreading globally, threatening our ability to treat common infectious diseases, which results in prolonged illness, disability, and death (World Health Organization, 2014). To date, many investigations have been focused on AR in bacteria found in humans, animals, and foods. AR genes are now considered emerging environmental contaminants due to their ability to be transferred to the environment from both humans and foods of vegetable (Thanner et al., 2016) and animal origin (He et al., 2016). Cox (2015) has recently suggested that the routine use of antibiotics in food-producing animals selects for antibiotic-resistant microorganisms that compromise human health, bringing us closer to a “post-antibiotic era” with multidrug-resistant “super-bugs.”

Insects can undoubtedly represent a source of antibiotic-resistant bacteria (Zurek and Ghosha, 2014). However, currently available data on the occurrence of antibiotic resistance in edible insects are very limited, with only a few published studies (Milanović et al., 2016; Osimani et al., 2017b,c; Vandeweyer et al., 2019) that deal with the distribution of selected AR genes in various edible insects. To the authors' knowledge, no investigations have been carried out to date to specifically correlate the occurrence of transferable AR genes in edible insects with their rearing conditions. Moreover, no studies have been carried out to determine the loads and dynamics of antibiotic resistance in specific microbial groups in edible insects.

Given this information, the distribution of AR genes in laboratory-reared fresh mealworm larvae (*Tenebrio molitor* L.), their feeding substrates (wheatmeal plus carrots as a water supplement), and frass (excrement from larvae mixed with substrate residues) was assessed. To this end, antibiotic-resistant microorganisms were enumerated onto selective growth media with antimicrobials. Moreover, the prevalence of 12 selected genes coding for resistance to antibiotics conventionally used in clinical practice such as MLS_B_ [*erm*(A), *erm*(B), *erm*(C)], vancomycin [*vanA, vanB*], tetracyclines [*tet*(M), *tet*(O), *tet*(S), *tet*(K)], β-lactams [*mecA, blaZ*] and amynoglicosides [*aac()-Ie aph(2*″*)-Ia*] (abbreviated as *aac-aph*) was determined using optimized PCR and nested PCR assays. The target genes were chosen among those that are most frequently detected in both human pathogens and commensal food-borne bacteria (Devirgiliis et al., 2011; Aarts and Margolles, 2015), which are widely recognized as potential reservoirs for these AR genes (Rolain, 2013). In parallel, the presence of 64 antibiotics that belong to 10 different drug families (amphenicols, beta-lactams, diamino-pyrimidine, lincosamides, macrolides, pleuromutilins, quinolones, rifamycins, sulfonamides, and tetracyclines) was assessed in the feed substrates.

## Materials and methods

### Insect rearing conditions

**Figure 1:**
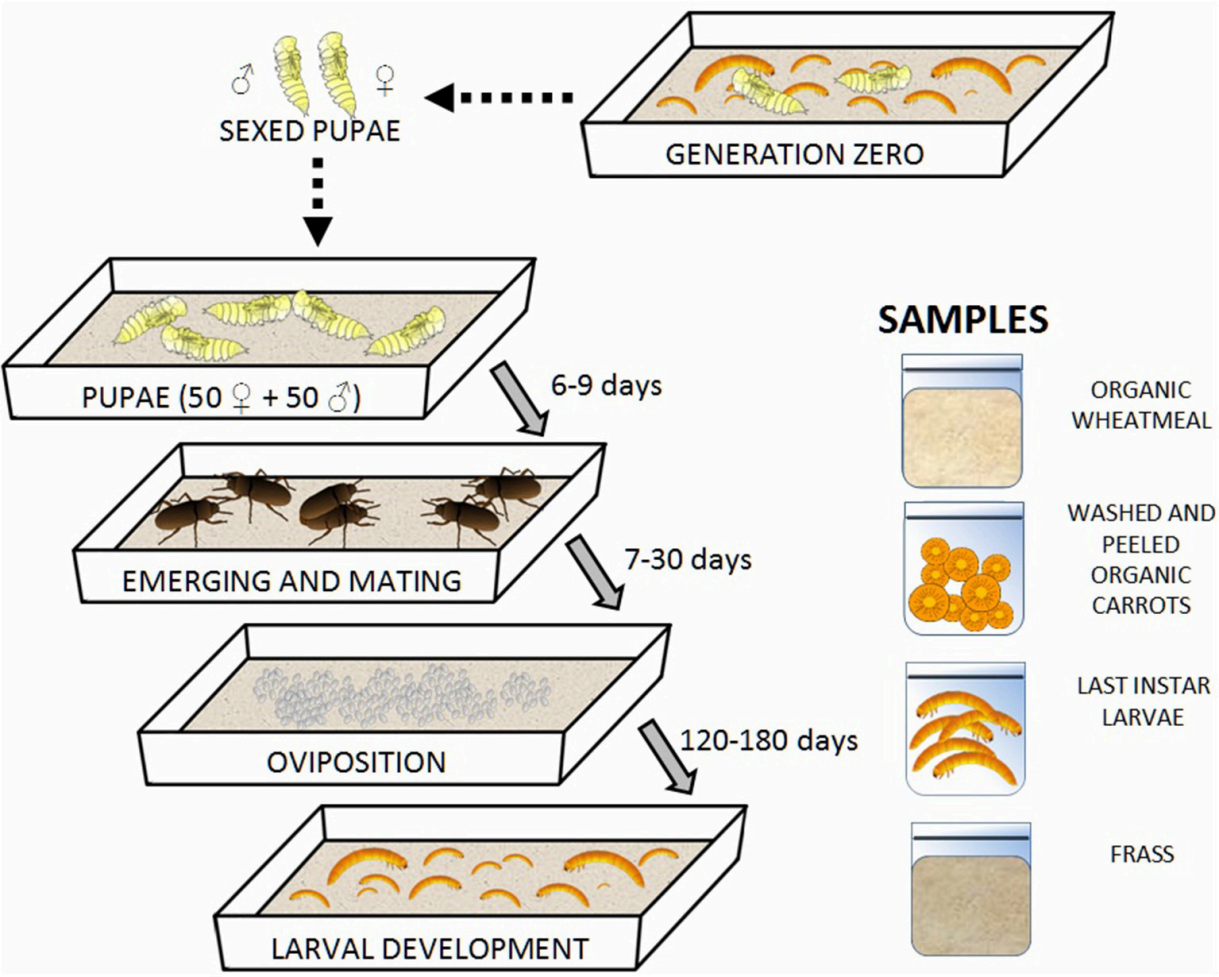
Rearing steps of mealworms and collected samples.

Mealworm larvae were purchased from a local pet store (Moby Dick, Jesi, Italy). They were grown in plastic boxes (21 x 30 x 6 cm) placed in a climate-controlled chamber at 28°C, 60% relative humidity (RH), and a 24-h dark photoperiod until the pupal stage was reached. Before use, the plastic boxes used for rearing were sanitized with a 3% active chlorine solution to avoid unwanted contamination. Organic wheatmeal, obtained from a local mill factory (Molino Agostini s.r.l, Osimo, Italy), and organic carrots (washed and peeled using sterile scalpels) were supplied as feed and water sources, respectively as suggested by Broekhovenv et al. (2015), Cortes Ortiz et al. (2016) and Dreassi et al. (2017) for the improvement of the insect growth. Pupae were sexed based on their morphology (Bhattacharya et al., 1970). Groups of 100 pupae, each including 50 females and 50 males, were placed in different plastic boxes containing fresh organic wheatmeal and carrots to allow adults to lay eggs. The second generation of larvae was grown under the same conditions as those described above. Three different batches of second-generation mealworms were reared in parallel. Figure 1 reports the rearing steps and the collected samples; the latter are described below. No antibiotics were used for the treatment of insects during rearing.

### Sampling

One batch of organic wheatmeal was aseptically sampled (~1 kg) prior to its use as feed for mealworms; aliquots of washed and peeled organic carrots (~100 g) used as a water supplement were also sampled under sterile conditions and pooled in one single sample. Carrots were not subjected to microbial counting because only the cores of the completely intact roots that were collected under sterile conditions have been administered to mealworms, thus assuring the absence of any microbial contamination.

Last instar larvae were individually collected from the three batches of second-generation mealworms and pooled together. Analogously, aliquots of frass were sampled from the three plastic boxes after collection of second-generation last instar larvae and pooled together for further analyses. The pooling of the samples was performed in order to reduce the effect of biological variation. All samples were collected into sterile plastic bags with sterile instruments.

### Bacterial enumeration

Ten grams of each pooled sample was homogenized in 90 mL of sterile peptone water (0.1% bacteriological peptone) at 260 rpm for 3 min using a Stomacher apparatus (400 Circulator, International PBI, Milan, Italy). Serial dilutions were prepared aseptically in peptone water, and 100 μL or 1-mL aliquots of each dilution were plated in duplicate onto agar plates for the enumeration of aerobic mesophilic bacteria, lactic acid bacteria (LAB), staphylococci (coagulase negative and positive), Pseudomonadaceae, enterococci and Enterobacteriaceae. For the enumeration of spore-forming bacteria, homogenates (10^−1^ dilution) of each sample were heat-shocked for 15 min at 80°C to inactivate the vegetative cells, then immediately cooled on ice, serially diluted in sterile peptone water and spread onto Plate Count Agar (PCA) (Milanović et al., 2017). The media, spreading method and the growth conditions are reported in Table 1.

**Table 1:** Culture media, incubation conditions and results of bacterial counts in wheatmeal used as feed, fresh mealworm larvae and frass of the three pooled rearing batches.

**Growth medium**	**Presumptive microorganism**	**Growth conditions**	**Plating method**	**Reference**	**Organic wheatmeal**	**Mealworm larvae**	**Frass**
					**log cfu g**^**−1**^		
PCA	Aerobic mesophilic bacteria	30°C;48 h	Pour	(American Public Health Association, 1976)	1.5 ± 0.08 ^c^	9.3 ± 0.04^a^	7.8 ± 0.01^b^
	Spore forming bacteria				<1^c^	2.3 ± 0.03 ^b^	4.1 ± 0.02^a^
MRS	Lactic acid bacteria	30°C;48–72 h	Pour	(De Man et al., 1960)	1.9 ± 0.08^c^	8.3 ± 0.01^a^	5.8 ± 0.01^b^
MRS + ampicillin (8 mg L^−1^)^*^	Ampicillin resistant lactic acid bacteria				<1^c^	2.2 ± 0.04^a^	1.7 ± 0.05^b^
MRS + vancomycin (8 mg L^−1^) ^*^	Vancomycin resistant lactic acid bacteria				1.3 ± 0.03 ^c^	7.1 ± 0.01^a^	3.9 ± 0.01^b^
ESA	Enterococci	37°C;24–48 h	Spread	(Slanetz and Bartley, 1957)	1.6 ± 0.07 ^c^	7.9 ± 0.01^a^	6.7 ± 0.03^b^
ESA + ampicillin (8 mg L^−1^) ^*^	Ampicillin resistant enterococci				<1^a^	<1^a^	<1^a^
ESA + gentamicin (128 mg L^−1^) ^*^	HLAR enterococci				<1^a^	<1^a^	<1^a^
ESA + vancomycin (4 mg L^−1^) ^*^	Vancomycin resistant enterococci				<1^c^	5.7 ± 0.03^a^	2.6 ± 0.01^b^
MSA	Staphylococci	37°C;24–48 h	Spread	(Chapman, 1945; Bannerman, 2003)	<1^b^	5.8 ± 0.01^a^	5.4 ± 0.01^a^
MSA + gentamicin (1 mg L^−1^) ^*^	Gentamicin resistant staphylococci				<1^b^	5.7 ± 0.01^a^	5.3 ± 0.01^a^
MSA + erythromycin (2 mg L^−1^) ^*^	Erythromycin resistant staphylococci				<1^c^	4.9 ± 0.04^a^	2.8 ± 0.10^b^
MSA + tetracycline (2 mg L^−1^)^*^	Tetracycline resistant staphylococci				<1^c^	5.2 ± 0.01^a^	4.6 ± 0.01^b^
MSA + vancomycin (2 mg L^−1^) ^*^	Vancomycin resistant coagulase positive staphylococci				<1^b^	4.1 ± 0.04^a^	4.3 ± 0.01^a^
MSA + vancomycin (4 mg L^−1^) ^*^	Vancomycin resistant coagulase negative staphylococci				<1^c^	3.8 ± 0.01^b^	4.3 ± 0.09^a^
VRBGA	Enterobacteriaceae	37°C;24 h	Pour	(American Public Health Association, 1976)	<1^c^	7.5 ± 0.01^a^	5.8 ± 0.01^b^
VRBGA + ampicillin (8 mg L^−1^) ^*^	Ampicillin resistant Enterobacteriaceae				<1^c^	6.1 ± 0.01 ^a^	5.0 ± 0.01 ^b^
VRBGA + gentamicin (4 mg L^−1^) ^*^	Gentamicin resistant Enterobacteriaceae				<1^c^	2.5 ± 0.01^a^	1.2 ± 0.20^b^
PAB	Pseudomonadaceae	30°C;24–48 h	Spread	(Geftic et al., 1979)	1.0 ±0.00^b^	6.7 ± 0.03^a^	6.1 ± 0.02^a^
PAB + gentamicin (4 mg L^−1^) ^*^	Gentamicin resistant Pseudomonadaceae				<1^c^	5.7 ± 0.03^a^	3.2 ± 0.01^b^

PCA, Plate Count Agar; MRS, de Man Rogosa Sharpe agar; ESA, Enterococcus Selective Agar, MSA, Mannitol Salt Agar; VRBGA, Violet Red Bile Glucose Agar; PAB, Pseudomonas Agar Base; HLAR, High-Level Aminoglycoside Resistance

* the breakpoint values defined by European Committee on Antimicrobial Susceptibility Testing (EUCAST, 2017)

Results of bacterial counts are expressed as mean values ± standard deviation of three independent experiments (one for each batch)

*Means followed by different letters are significantly different (P < 0.05)*.

For the viable counts of antibiotic-resistant bacteria that were present in the analyzed samples, the media listed in Table 1 were supplemented with concentrations of ampicillin, gentamicin, erythromycin, tetracycline and vancomycin based on the breakpoint values defined by the European Committee on Antimicrobial Susceptibility Testing (EUCAST, 2017) for each group of analyzed bacteria (Table 1). The results of bacterial enumeration were expressed as the mean ± standard deviation of the log of colony-forming units (cfu) per gram of sample.

### Detection of antibiotic residues in organic wheatmeal and carrots

The analysis of the 64 antibiotics listed in Table 2, which belong to ten drug families (amphenicols, beta-lactams, diamino-pyrimidines, lincosamides, macrolides, pleuromutilins, quinolones, rifamycins, sulfonamides and tetracyclines), was carried out in wheatmeal and pooled carrots as described by Moretti et al. (2016) with some modifications regarding the sample preparation protocol of the wheatmeal. Briefly, 2 g of organic wheatmeal was sequentially extracted using acetonitrile/water 50/50 (v/v), acetonitrile containing formic acid at 0.1%, and an aqueous solution of 0.1 M Na_2_EDTA. An aliquot was collected from each extract and placed into a tube. After evaporation, the extract was redissolved in ammonium acetate buffer prior to injection. For testing, a Thermo Ultimate 3,000 Ultra High-Performance Liquid Chromatography system coupled with a high-resolution Q-Exactive mass spectrometer (Thermo Scientific, San Jose, CA, USA) operating in positive electrospray ionization mode was used. Chromatographic separation was performed with a Poroshell 120 EC-C18 column (100 × 3.0 mm, 2.7 μm) using gradient elution (mobile phases: methanol and water containing 0.1% of formic acid). The acquisition mode was fully scanned with some analytes in t-SIM to overcome the strong matrix effects in wheatmeal. The results were expressed as μg of antibiotic per kg of food matrix (μgkg^−1^ or ppb) with detection limits equal to 100 and 10 μgkg^−1^ (cefacetrile: 33 μgkg^−1^) for wheatmeal and carrots, respectively.

**Table 2:** List of the 64 antibiotics determined in wheatmeal and carrots used as feed.

**#**	**Antibiotic**	**#**	**Antibiotic**
1	Thiamphenicol	33	Erithromycin A
2	Florfenicol	34	Spiramycin I
3	Florfenicol Amine	35	Neospiramycin
4	Cephapirin	36	Tylosin A
5	Desacetylcephapirin	37	Tilmicosin
6	Ceftiofur	38	Tulathromycin
7	Cefalexin	39	Tulathromycin marker
8	Cefquinome	40	Tildipirosin
9	Cefazolin	41	Tylvalosin (acetyl-isovaleryltyrosine)
10	Cefoperazone	42	3-O-Acetyltylosin
11	Cefalonium	43	Gamithromycin
12	Cefacetrile	44	Tiamulin
13	Penicillin G	45	Valnemulin
14	Amoxicillin	46	Rifaximin
15	Ampicillin	47	Sulfanilamide
16	Cloxacillin	48	Sulfamethazine
17	Dicloxacillin	49	Sulfapyridine
18	Oxacillin	50	Sulfadiazine
19	Nafcillin	51	Sulfadimethoxine
20	Penicillin V	52	Sulfamonomethoxine
21	Difloxacin	53	Sulfaquinoxaline
22	Flumequine	54	Sulfathiazole
23	Oxolinic Acid	55	Sulfaguanidinae
24	Ciprofloxacin	56	Sulfamerazine
25	Enrofloxacin	57	Sulfamethoxazole
26	Danofloxacin	58	Chlortetracycline
27	Marbofloxacin	59	Epichlorotetracycline
28	Sarafloxacin	60	Oxitetracyclin
29	Nalidixic Acid	61	Epioxitetracyclin
30	Norfloxacin	62	Doxycycline
31	Trimethoprim	63	Tetracyclin
32	Lincomycin	64	Epitetracycline

### Reference strains

Twelve antibiotic-resistant bacterial strains, each carrying one AR gene of interest, were used as positive controls in the PCR and nested PCR reactions (Table 3). *Enterococcus faecalis* JH2-2 (Jacob and Hobbs, 1974), free of AR genes under study, was used as a negative control.

**Table 3:** Bacterial reference strains used in this study.

**AR gene**	**Gene function**	**Strains positive for AR genes**
*erm*(A)	rRNA methylase gene	*Staphylococcus aureus* M.P. ^a^
*erm*(B)	rRNA methylase gene	*Enterococcus hirae* Api 2.16 ^a^
*erm*(C)	rRNA methylase gene	*Staphylococcus* spp. SE12 ^b^
*vanA*	Peptidoglycan precursors encoding gene	*Enterococcus faecium* PF3U ^b^
*vanB*	Peptidoglycan precursors encoding gene	*Enterococcus faecalis* ATCC 51299 ^c^
*tet*(M)	Ribosomal protection RP gene	*Lactobacillus casei/paracasei* ILC2279 ^b^
*tet*(O)	Ribosomal protection RP gene	*Streptococcus pyogenes* 7008 ^a^
*tet*(S)	Ribosomal protection RP gene	*Enterococcus italicus* 1102 ^b^
*tet*(K)	Efflux gene	*Staphylococcus aureus* COL. ^a^
*mecA*	β-lactamase encoding gene	*Staphylococcus aureus* 27R ^b^
*blaZ*	β-lactamase encoding gene	*Staphylococcus aureus* ATCC 2921 ^c^
*aac-aph*	Aminoglycoside acetyltransferase encoding gene	*Enterococcus faecium* M48 ^a^

a *Collection of Dipartimento di Scienze della Vita e dell'Ambiente (DiSVA), Università Politecnica delle Marche, Italy*.

b *Collection of Dipartimento di Scienze Agrarie, Alimentari ed Ambientali (D3A), Università Politecnica delle Marche, Italy*.

c *ATCC, American Type Culture Collection*.

### DNA extraction

The DNA from the 12 reference strains and the negative control strain *E. faecalis* JH2-2 was extracted as described by Hynes et al. (1992), with some modifications as reported by Osimani et al. (2015). The metagenomic DNA from organic wheatmeal, pooled samples of peeled organic carrots, second-generation mealworm larvae, and frass was extracted using the PowerFood Microbial DNA Isolation Kit (Mo Bio Laboratories, Carlsbad, United States) as previously described by Milanović et al. (2016). The organic wheatmeal and the carrots were subjected to DNA extraction prior to their use as a feed and a water supplement. The DNA extracts were assessed for quantity and purity by optical readings at 260, 280, and 234 nm using a UV-Vis Shimadzu UV-1800 spectrophotometer (Shimadzu, Kyoto, Japan).

### PCR and nested PCR amplification of AR genes

DNA extracts obtained from wheatmeal and each pooled sample (carrots, mealworms, and frass) were amplified in PCR assays targeting *erm*(A), *erm*(B), *erm*(C), *tet*(M), *tet*(O), *tet*(S), *tet*(K), *vanA, vanB, blaZ, mecA*, and *aac-aph* genes. The specific mechanisms of resistance for these genes are reported in Table 3. The samples that were negative following the first round of PCR were further subjected to nested PCR. Amplification conditions and primers used for PCR and nested PCR were previously described by Garofalo et al. (2007) and Milanović et al. (2016) and are reported in Tables S1, S2.

Two microliters of DNA extract (containing ~10 ng of microbial DNA) or PCR product was amplified by PCR or nested PCR, respectively, in a total volume of 25 μL under the same conditions reported by Osimani et al. (2017b). Briefly, the reaction mixture contained the following: 1X buffer, 50 pmol of each primer, 0.2 mM of dNTPs (2.5 mM for the amplification of *erm* genes in both assays), and 0.75 U of Taq polymerase. Positive (Table 3) and negative (*E. faecalis* JH2-2) controls were used in each PCR assay. PCR mixture supplemented with water instead of DNA was used as a blank. All amplifications were carried out in a MyCycler thermal cycler (Bio-Rad Laboratories, Hercules, United States). Five microliters of each PCR product was analyzed by electrophoresis through a 1.5% (w/v) agarose gel (Conda pronadisa, Spain) in 0.5X TBE (45 mM Tris-borate, 1 mM EDTA) containing 0.5 μgmL^−1^ ethidium bromide at 75 V for 45 min. A 100-bp DNA Ladder (SibEnzyme Ltd., Academtown, Russia) was used as a molecular weight standard. A Complete Photo XT101 system (Explera, Jesi, Italy) was used to visualize gels under UV light. For each tested AR gene, randomly selected PCR products were sequenced by Genewiz (Hope End, Takeley, United Kingdom) to verify the annealing of oligos to the proper target sequences.

### Statistical analysis

Microbial count data were subjected to one-way analysis of variance (ANOVA) carried out using JMP statistical software version 11.0.0 (SAS Institute Inc., NC, United States). Differences were considered significant at *P* < 0.05.

## Results and discussion

In this study, a PCR-based metagenomic approach has been applied to investigate the distribution of 12 selected AR genes in a laboratory scale *T. molitor* rearing cycle. The novelty of this approach relies on the possibility of evaluating the occurrence of AR under controlled rearing conditions without any selective pressure exerted by antibiotics. Such an approach also allowed to exclude any possible interference of insect manipulation and/or processing on the occurrence of transferable AR genes and antibiotic-resistant microorganisms in edible insects, thus constituting an advancement on the knowledge in respect with the available literature on the same topic (Milanović et al., 2016; Osimani et al., 2017b,c; Vandeweyer et al., 2019).

Among edible insects, *T. molitor* undoubtedly represents a very promising food source being very rich in protein and fat and easy to breed (Gasco et al., 2016; Zhao et al., 2016; Osimani et al., 2017a). It is also a good source of polyunsaturated fatty acids whose consumption is considered a potential means of improving health (Mozzon et al., 2002; Haddad et al., 2012; Pacetti et al., 2013). Therefore, its potential use in the food industry can rapidly increase (Stoops et al., 2017).

The results of the viable counts of aerobic mesophilic bacteria, spore-forming bacteria, lactic acid bacteria, enterococci, staphylococci, Enterobacteriaceae and Pseudomonadaceae assessed in this study are reported in Table 1. To the authors' knowledge, this is the very first study on the enumeration of antibiotic resistant microorganisms in edible insects as revealed by viable plate counting.

As a general trend, viable counts of aerobic mesophilic bacteria in wheatmeal were in the range of those reported by Alfonzo et al. (2017) for whole-meal semolinas collected from different Italian regions, which ranged between <1 and 4.0 ± 0.3 log cfu g^−1^. Even in the pooled samples of mealworm larvae and frass, the load of this microbial group was similar to that found in the same matrices by Vandeweyer et al. (2017) and Wynants et al. (2017), respectively, with the latter reporting viable counts between 8.8 and 11.4 log cfu g^−1^.

For spore-forming bacteria, viable counts in the feed were again similar to those reported by Valerio et al. (2012) in semolina, with loads ranging between <1 and 2 log cfu g^−1^. This bacterial group is commonly found in cereals and cereal-based matrices. It is noteworthy that spore-forming bacterial species can represent the causative agents of food-borne intoxication because of their toxin-forming ability. Regarding mealworms, these microbial group counts were again in the ranges reported by different authors for the same insect species (Klunder et al., 2012; Stoops et al., 2016, 2017; Vandeweyer et al., 2017).

LAB counts in wheatmeal were similar to those recorded in whole-meal semolinas by Alfonzo et al. (2017), ranging between <1 and 3.5 ± 0.4 log cfu g^−1^. It is known that LAB contamination is very common in cereal grains where their occurrence can be both endophytic or environmental (Alfonzo et al., 2013). In fresh larvae, LAB showed an average load of 8.3 log cfu g^−1^ that was in the range of the values reported by both Stoops et al. (2016) and Vandeweyer et al. (2017) for larvae of the same species, where these microorganisms had an average value of about 7 log cfu g^−1^.

Regarding the LAB counts, notably higher mean values were seen in the mealworm larvae with respect to wheatmeal or frass. Different pictures emerged when considering the loads of ampicillin- and vancomycin-resistant LAB. Indeed, in all of the samples analyzed, the viable counts of LAB on MRS with ampicillin were significantly lower than those on the same medium with no antibiotics. In more detail, in organic wheatmeal, no LAB resistant to ampicillin were detected (<1 log cfu g^−1^), whereas in larvae and frass, the counts of ampicillin-resistant LAB were 6 and 4 orders of magnitude lower, respectively. As reviewed by Clementi and Aquilanti (2011), ampicillin resistance is poorly documented in LAB; however, resistant strains in this microbial group have occasionally been isolated from foods of animal origin, including *Lactobacillus plantarum* from raw meat (Aquilanti et al., 2007) or *Enterococcus* spp. from milk and cheese (Riboldi et al., 2009). Interestingly, members of the genus *Enterococcus* have already been found in the microbiota of edible insects and in both dried and fresh mealworms (Garofalo et al., 2017; Vandeweyer et al., 2017; Wynants et al., 2017).

Regarding the LAB counts on MRS with vancomycin, bacterial loads in wheatmeal were on the same order of magnitude as those assessed on MRS without antibiotic (about 2 log cfu g^−1^); in contrast, the loads of vancomycin-resistant LAB in mealworm larvae and frass were one and two orders of magnitude lower, respectively. Among LAB, foodborne leuconostocs, pediococci and most lactobacilli species are known to be intrinsically resistant to vancomycin, whereas the majority of lactococci and enterococci are susceptible to this antibiotic (Clementi and Aquilanti, 2011).

As far as enterococci are concerned, neither ampicillin-resistant nor high-level aminoglycoside-resistant (HLAR) enterococci were enumerated in the analyzed samples (loads <1 log cfu g^−1^), despite the high counts of this microbial group on Enterococcus Selective Agar (ESA) medium. Conversely, vancomycin-resistant enterococci were identified in both mealworm larvae and frass, with average viable counts of 5.7 and 2.6 log cfu g^−1^, respectively. Antibiotic-resistant enterococci have a great clinical importance due to the contribution of these microorganisms to nosocomial infection risk. To date, several reports described the occurrence of foodborne enterococci that are highly resistant to vancomycin (Riboldi et al., 2009), thus supporting the need to deepen our knowledge about the distribution of these microorganisms in novel foods.

When the counts of staphylococci are considered, there were no viable colonies from wheatmeal on Mannitol Salt Agar (MSA) medium. In contrast, larvae and frass showed comparable counts at about 5–6 log cfu g^−1^. To the authors' knowledge, there is a scarcity of available information on staphylococci counts in edible insects. Very recently, Wynants et al. (2017) found viable coagulase-positive staphylococci below 2 log cfu g^−1^ in fresh larvae of *Alphitobius diaperinus* intended for human consumption. A few years before, Oliveira et al. (2014) isolated pathogenic strains of *Staphylococcus aureus* in several live insects (ants, cockroaches, flies, wasps, gnats, moths and butterflies) from Brazilian hospitals.

In the present study, staphylococci resistant to all assayed antibiotics were enumerated in all of the analyzed samples except wheatmeal. As a general trend, mean counts between 2.8 and 5.7 cfu g^−1^ were observed, with erythromycin-resistant staphylococci in frass having the lowest mean counts and gentamicin-resistant staphylococci in larvae having the highest. As reported by Haaber et al. (2017), staphylococci, including pathogenic *S. aureus*, can readily adapt to changing environments and can acquire AR genes through different mechanisms. Although the method by which transfer occurs *in vivo* is still unclear, transduction and conjugation are considered the most prevalent mechanisms. Although antibiotic-resistant staphylococci have already been isolated from a number of animal-based foods (Chajecka-Wierzchowska et al., 2015; Fijałkowski et al., 2016), no reports on the occurrence of AR in staphylococci from edible insects are yet available.

Concerning Enterobacteriaceae in wheatmeal, no growth was seen on both Violet Red Bile Glucose Agar (VRBGA) and VRBGA with ampicillin or gentamicin. As a general trend, higher counts were seen in larvae with respect to frass, irrespective of the growth medium used. For the loads of Enterobacteriaceae on VRBGA, our results are in accordance with those of Vandeweyer et al. (2017) and Wynants et al. (2017) analyses of fresh industrially reared *T. molitor* larvae.

Finally, regarding Pseudomonadaceae, in wheatmeal plated on Pseudomonas Agar Base (PAB), mean counts of 1 log cfu g^−1^ were detected. These microorganisms, and particularly members of the genus *Pseudomonas*, are known to exert plant-promoting activity in the wheat rhizosphere (Godino et al., 2016), thus explaining their presence in wheatmeal used as feed. Higher mean values were seen in both larvae and frass at about 6 log cfu g^−1^ in respect with wheatmeal. Concerning viable counts on PAB with gentamicin, wheatmeal had mean counts below 1 log cfu g^−1^, whereas larvae and frass had significantly higher mean values at 5.7 and 3.2 log cfu g^−1^, respectively. Notoriously, *Pseudomonas* includes both spoilage species and opportunistic pathogens (e.g., *Pseudomonas aeruginosa*), which have previously been detected in insects such as cockroaches, grasshoppers and lygaeid bugs (Grabowski and Klein, 2017).

In the present study, the DNA extracted directly from the pooled samples of fresh mealworm larvae, feed substrates (wheatmeal and pooled carrots), and frass collected from a laboratory-scale production chain of *T. molitor* was analyzed by using a PCR-based approach. In more detail, samples negative after first round of amplification reactions were further subjected to nested PCR with an internal primer set, which was specifically conceived to hybridize to secondary internal targets within the main amplified regions. This allows for more sensitive detection of the targeted genes, ranging from 10^0^ [*tet*(M), *erm*(C), *vanA, blaZ*] to 10^3^ [*erm*(A)] copies per sample, depending on the gene (Garofalo et al., 2007). In accordance with the results obtained in previous studies (Milanović et al., 2016; Osimani et al., 2017b,c), the enhanced sensitivity of nested PCR allowed for increased detection of most of the target AR genes.

Regarding fresh larvae, *erm*(A), *erm*(C), *vanA, vanB, tet*(O), *mecA, blaZ*, and *aac-aph* were absent in all of the samples.

In contrast, mealworms carried the tetracycline resistance genes *tet*(M), *tet*(K), and *tet*(S) and the MLS_B_ resistance gene *erm*(B) (Table 4).

**Table 4:** Results of PCR and nested PCR amplification of AR genes in samples of carrots and wheatmeal used as feed, and fresh mealworm larvae and frass of the three pooled rearing batches.

**Sample**	**Assay**	**Antibiotic resistance gene**											
		***erm*(A)**	***erm*(B)**	***erm*(C)**	***vanA***	***vanB***	***tet*(M)**	***tet*(O)**	***tet*(S)**	***tet*(K)**	***mecA***	***blaZ***	***aac-aph***
Organic carrots	PCR	–	–	–	–	–	–	–	–	–	–	–	–
	*n*-PCR	–	–	–	–	–	–	–	–	–	–	–	–
Organic wheatmeal	PCR	–	–	–	–	–	–	–	–	–	–	–	–
	*n*-PCR	–	–	–	–	–	–	–	+	+	–	–	–
Mealworm larvae	PCR	–	–	–	–	–	–	–	+	–	–	–	–
	*n*-PCR	–	+	–	–	–	+	–	n.d.	+	–	–	–
Frass	PCR	–	–	–	–	–	–	–	+	–	–	–	–
	*n*-PCR	–	+	–	–	–	+	–	n.d.	+	–	–	+

*PCR, positive after PCR; n-PCR: positive after nested PCR; n.d. not determined*.

Due to their wide-ranging spectrum of activity, tetracyclines represent one of the most widely used classes of antibiotics in clinics. In Gram-negative bacteria, tetracycline resistance mechanisms involve efflux pump systems that are encoded by different tetracycline resistance genes (Hwang et al., 2017). As reported by the ECDC, EFSA, and EMA, significant positive associations were observed between tetracycline AR in human cases of salmonellosis and campylobacteriosis and in tetracycline consumption in animals in Europe, with a possible role in co-selection through the genetic linkage of resistance genes (ECDC/EFSA/, 2015).

There is still a lack of knowledge regarding AR associated with edible insects. Very recently, Osimani et al. (2017b,c) found a high distribution of *tet*(M), *tet*(K), and *tet*(S) in ready-to-eat grasshoppers and mealworms purchased from both European and non-European producers. Moreover, high detection frequencies of the same tetracycline resistance genes have previously been reported by Milanović et al. (2016) in commercially available dried mealworms. In a few investigations, tetracycline-resistant isolates were collected from various insect-based matrices. In more detail, Usui et al. (2015) isolated *tet*^R^ *Escherichia coli* strains from both flies and feces of livestock from different farms, suggesting a role for these insects in spreading transferable resistance among the different farms under investigation. A few years before, Larson et al. (2008) found that insects associated with stored feed could be vectors of tetracycline-resistant enterococci.

Macrolides are one of the most important antibiotics used in the clinical treatment of various illnesses, including community-acquired pneumonia, infections caused by *Shigella* and *Salmonella*, and sexually transmitted diseases. Bacterial resistance to this class of antibiotics is determined by an erythromycin ribosomal methyltransferase gene (Fyfe et al., 2016). Regarding resistance to macrolides, the results obtained in the present study agree with those reported by Milanović et al. (2016) and Osimani et al. (2017b) for ready-to-eat insects. Furthermore, Osimani et al. (2017c) recently found resistance to erythromycin in samples of dried edible mealworms ready for consumption that are produced in both European and non-European countries. In more detail, Osimani et al. (2017c) found a very high occurrence of *erm*(B)-positive samples in mealworms produced in France. To the authors' knowledge, no other studies on erythromycin resistance in insects are presently available in the scientific literature.

In frass *tet*(M), *tet*(K), *tet*(S), and *aac-aph* were detected (Table 4). Aminoglycosides are normally used as broad-spectrum antibiotics against aerobic Gram-negative bacteria. Resistance to this class of antibiotics, which includes streptomycin, neomycin, kanamycin, gentamicin, and tobramycin, can be exerted via N-acetylation, O-nucleotidylation, and/or O-phosphorylation inactivation and efflux pumps (Sheikhalizadeh et al., 2017). In 2008, Larson et al. first isolated strains of *Enterococcus gallinarum* from maize weevil (*Sitophilus zeamais* Motschulsky) and the warehouse beetle (*Trogoderma variabile* Ballion) that were resistant to streptomycin and neomycin, while more recently, Osimani et al. (2017b,c) detected resistance genes to this class of antibiotics in commercially available ready-to-eat grasshoppers and mealworms.

Although in the present study no vancomycin resistance genes were detected, it is worth noting that Osimani et al. (2017c) have recently found *vanA-* and *vanB*-positive samples in ready-to-eat mealworms collected from European producers located in France and Belgium. These findings are particularly important since the increasing emergence of vancomycin-resistant *Enterococcus faecium* strains has recently been reported by the EFSA/ECDC (2016).

Insect frass is a minimally investigated rearing waste, especially in terms of the occurrence of transferable AR. As recently reported by He et al. (2016), rearing wastes can result in environmental contamination by AR genes, leading to potential risks to food safety and human health. This hypothesis was also supported by other studies, which demonstrated that the levels of genes conferring resistances to tetracyclines and MLS_B_ remained high in animal wastes during composting, lagoon storage, anaerobic digestion, and wetland construction (Wang et al., 2012; Brooks et al., 2014; Huang et al., 2014; Tao et al., 2014).

The results of the quantitative assessment of antibiotic residues in organic wheatmeal and carrots are reported in Table 2. The choice to analyze these substrates was driven by the results of a meta-analysis revealing that even organic produce, including vegetables, can effectively be contaminated with pesticides and antibiotic residues, with the latter potentially exerting a selective pressure on the resident microbiota (Smith-Spangler et al., 2012). None of the screened antibiotics were found in the two feed substrates, considering the limits of detection of 100 and 10 μgkg^−1^ (cefacetrile: 33 μgkg^−1^) for wheatmeal and carrots, respectively.

Regarding the screened AR genes, organic wheatmeal was found to carry both *tet*(S) and *tet*(K) (Table 4), whereas no AR genes were detected in organic carrots. Although the microorganisms contaminating the organic wheatmeal used as feed presumably were not subjected to selective pressure by antibiotics, Lin and Kussel (2016) reported that the occurrence of AR is highly dependent on the antibiotic exposure dynamics. The same authors demonstrated that the physiological memory of microbial cells can strongly modulate the emergence of resistance (Lin and Kussel, 2016). In a relatively recent study, Fernández-Fuentes et al. (2012) detected multi-resistant microbial strains in organic foods, including strains of *Chryseobacterium* sp. resistant to erythromycin, *Enterobacter* resistant to amoxicillin and cefuroxime, *E. casseliflavus* resistant to amoxicillin and erythromycin, *Enterococcus faecium* resistant to amoxicillin and erythromycin, and *Pantoea agglomerans* resistant to cefuroxime.

## Conclusions

As a general trend, lower bacterial counts were ascertained in wheatmeal compared to the larvae and frass. On the one hand, this might suggest that the microorganisms originating from wheatmeal colonize and multiply in the gut of larvae. On the other hand, the vertical transmission of microorganisms could have contributed to both the composition and quantity of microorganisms in the larvae and frass (Engel and Moran, 2013; Itoh et al., 2014; Shapira, 2016).

Moreover, the occurrence of *tet*(S) and *tet*(K) in organic wheatmeal, larvae and frass suggests the effective role of feed in the occurrence of AR genes and/or antibiotic resistant microorganisms in larvae, under no selective pressure exerted by antibiotics. In addition, the fact that larvae and frass were found to carry AR genes that were not detectable in organic wheatmeal seems to confirm that AR genes or antibiotic-resistant microorganisms (e.g., symbionts) can be transferred vertically via insect egg smearing.

AR in bacteria associated with food animals represents a risk to human health. In light of the use of edible insects as a new source of protein, the evaluation of AR within the production chain of this novel food constitutes a step forward in risk assessment.

The results overall collected by analyzing fresh larvae reared in a laboratory facility under no selective pressure exerted by antibiotics (either used as growth promoters or emergency treatment or even occurring as residues in feed) suggest that edible insects can be natural carrier of AR.

## Author contributions

VM, FeC, and CG carried out microbiological and molecular analyses. SR, PR, NI, and NL reared the edible insects under study. RG, SM, AP, EM, and FT carried out chemical analyses on insects and substrates. AO, LA, and FrC conceived the research, critically analyzed the results and wrote the manuscript.

We wish to thank Dr. Rico Marabini for his valuable support with the molecular analyses.

### Conflict of interest statement

The authors declare that the research was conducted in the absence of any commercial or financial relationships that could be construed as a potential conflict of interest.
