# Supplementary material for: Distribution of Transferable Antibiotic Resistance Genes in Laboratory-Reared Edible Mealworms (Tenebrio molitor L.)
Source: Front Microbiol. 2018 Nov 19;9:2702. doi: 10.3389/fmicb.2018.02702 (PMC6252353; doi:10.3389/fmicb.2018.02702)
Supplement: Supplementary file 1 [file Table_1.DOC]

**Table S1** Primers and PCR conditions

| **Primer** | **Sequence** | **PCR conditions** |  |  |
| --- | --- | --- | --- | --- |
|  |  | **Initial denaturation** | **Cycle** | **Final extension** |
| *aac(6’)-Ie-*  *aph(2”)-Ia* | 1-GAGCAATAAGGGCATACCAAAAATC  2- CCGTGCATTTGTCTTAAAAAACTGG | 95 °C for 5 min | 35 cycles of denaturation at 94 °C for 30 sec, annealing at 58 °C for 30 sec and extension at 72 °C for 30 sec | 72 °C for 7 min |
| *erm*(A) | 1-CAGGAAAAGGACATTTTACCAA  2-CTTCGATAGTTTATTAATATTAGT | 95 °C for 5 min | 35 cycles of denaturation at 94 °C for 1 min, annealing at 50 °C for 1 min and extension at 72 °C for 1 min | 72 °C for 7 min |
| *erm*(B) | 1-GAAAAGGTACTCAACCAAATA  2-AGTAACGGTACTTAAATTGTTTAC | 95 °C for 5 min | 35 cycles of denaturation at 94 °C for 1 min, annealing at 54 °C for 1 min and extension at 72 °C for 1 min | 72 °C for 7 min |
| *erm*(C) | 1-TCAAAACATAATATAGATAAA  2-GCTAATATTGTTTAAATCGTCAAT | 95 °C for 5 min | 35 cycles of denaturation at 94 °C for 1 min, annealing at 50 °C for 1 min and extension at 72 °C for 1 min | 72 °C for 7 min |
| *vanA* | 1-GGGAAAACGACAATTGC  2-GTACAATGCGGCCGTTA | 95 °C for 5 min | 35 cycles of denaturation at 94 °C for 30 sec, annealing at 58 °C for 30 sec and extension at 72 °C for 30 sec | 72 °C for 7 min |
| *vanB* | 1-ATGGGAAGCCGACAGTC  2-GATTTCGTTCCTCGACC | 95 °C for 5 min | 35 cycles of denaturation at 94 °C for 30 sec, annealing at 58 °C for 30 sec and extension at 72 °C for 30 sec | 72 °C for 7 min |
| *tet*(M) | 1-ACCCGTATACTATTTCATGCACT  2-CCTTCCATAACCGCATTTTG | 95 °C for 5 min | 35 cycles of denaturation at 94 °C for 1 min, annealing at 48 °C for 1 min and extension at 72 °C for 1 min | 72 °C for 7 min |
| *tet*(O) | 1-AACTTAGGCATTCTGGCTCAC  2-TCCCACTGTTCCATATGCTCA | 95 °C for 5 min | 35 cycles of denaturation at 94 °C for 1 min, annealing at 62 °C for 1 min and extension at 72 °C for 1 min | 72 °C for 7 min |
| *tet*(S) | 1-TTCCTTTGGGTAGTGGCAT^b^  2-ACAACGGGCTGGAATTTCAC^b^ | 95 °C for 5 min | 35 cycles of denaturation at 94 °C for 1 min, annealing at 60 °C for 1 min and extension at 72 °C for 1 min | 72 °C for 7 min |
| *tet*(K) | 1-TCGATAGGAACAGCAGTA  2-CAGCAGATCCTACTCCTT | 95 °C for 5 min | 35 cycles of denaturation at 94 °C for 30 sec, annealing at 55 °C for 30 sec and extension at 72 °C for 30 sec | 72 °C for 7 min |
| *mec*A | 1-GGGATCATAGCGTCATTATTG  2-AGTTCTGCAGTACCGGATTTGC | 95 °C for 5 min | 35 cycles of denaturation at 94 °C for 30 sec, annealing at 58 °C for 30 sec and extension at 72 °C for 30 sec | 72 °C for 7 min |
| *bla*Z | 1-ACTTCAACACCTGCTGCTTTC  2-TAGGTTCAGATTGGCCCTTAG | 95 °C for 5 min | 35 cycles of denaturation at 94 °C for 30 sec, annealing at 58 °C for 30 sec and extension at 72 °C for 30 sec | 72 °C for 7 min |

**Table S2** Primers and nested PCR conditions

| **Primer** | **Sequence** | **PCR conditions** |  |  |
| --- | --- | --- | --- | --- |
|  |  | **Initial denaturation** | **Cycle** | **Final extension** |
| *aac(6’)-Ie-aph(2”)-Ia* | 1-GAGCAATAAGGGCATACCAAAAATC  4- GCCACACTATCATAACCACT | 95 °C for 5 min | 35 cycles of denaturation at 94 °C for 30 sec, annealing at 62 °C for 30 sec and extension at 72 °C for 30 sec | 72 °C for 7 min |
| *erm*(A) | 1-CAGGAAAAGGACATTTTACCAA  3-CTATAGAAATTGATGGAGGCTTA | 95 °C for 5 min | 35 cycles of denaturation at 94 °C for 1 min, annealing at 58 °C for 1 min and extension at 72 °C for 1 min | 72 °C for 7 min |
| *erm*(B) | 3-CAATTCCCTAACAAACAGAGG  2-AGTAACGGTACTTAAATTGTTTAC | 95 °C for 5 min | 35 cycles of denaturation at 94 °C for 30 sec , annealing at 60 °C for 30 sec and extension at 72 °C for 30 sec | 72 °C for 7 min |
| *erm*(C) | 3-GTAATTTCGTAACTGCCATT  4-GCATGTTTTAAGGAATTGTT | 95 °C for 5 min | 35 cycles of denaturation at 94 °C for 30 sec , annealing at 52 °C for 30 sec and extension at 72 °C for 30 sec | 72 °C for 7 min |
| *van*A | 3-GTAGGCTGCGATATTCAAAGC  4-CGATTCAATTGCGTAGTCCAAT | 95 °C for 5 min | 35 cycles of denaturation at 94 °C for 30 sec, annealing at 58 °C for 30 sec and extension at 72 °C for 30 sec | 72 °C for 7 min |
| *van*B | 3-GGTGCGATACAGGGTCTGTT  4-GGAATGTCTGCTGGAACGAT | 95 °C for 5 min | 35 cycles of denaturation at 94 °C for 30 sec, annealing at 58 °C for 30 sec and extension at 72 °C for 30 sec | 72 °C for 7 min |
| *tet*(M) | 3-CTTAGGAAAATGGGGATTCC  4-GCGGTGATACAGATAAACC | 95 °C for 5 min | 35 cycles of denaturation at 94 °C for 30 sec, annealing at 50 °C for 30 sec and extension at 72 °C for 30 sec | 72 °C for 7 min |
| *tet*(O) | 3-TACCAGTGGTGCAATTGCAGA  4-TTATATGGGGATGCTGCCCAA | 95 °C for 5 min | 35 cycles of denaturation at 94 °C for 30 sec, annealing at 58 °C for 30 sec and extension at 72 °C for 30 sec | 72 °C for 7 min |
| *tet*(S) | 3-CGCTATGGGTGTGAACAAGG^b^  4-GGAAATCTGCTGGCGTACTG^b^ | 95 °C for 5 min | 35 cycles of denaturation at 94 °C for 30 sec, annealing at 64 °C for 30 sec and extension at 72 °C for 30 sec | 72 °C for 7 min |
| *tet*(K) | 3-GAACAGCAGTATATGGAA  4-AAAAAGTGATTGTGACCA | 95 °C for 5 min | 35 cycles of denaturation at 94 °C for 30 sec, annealing at 50 °C for 30 sec and extension at 72 °C for 30 sec | 72 °C for 7 min |
| *mec*A | 3-AAAATCGATGGTAAAGGTTGGC  2-AGTTCTGCAGTACCGGATTTGC | 95 °C for 5 min | 35 cycles of denaturation at 94 °C for 30 sec, annealing at 55 °C for 30 sec and extension at 72 °C for 30 sec | 72 °C for 7 min |
| *bla*Z | 1-ACTTCAACACCTGCTGCTTTC  4-TGACCACTTTTATCAGCAACC | 95 °C for 5 min | 35 cycles of denaturation at 94 °C for 30 sec, annealing at 58 °C for 30 sec and extension at 72 °C for 30 sec | 72 °C for 7 min |
